# Early relapse on adjuvant gemcitabine associated with an exceptional response to 2nd line capecitabine chemotherapy in a patient with pancreatic adenosquamous carcinoma with strong intra-tumoural expression of cytidine deaminase: a case report

**DOI:** 10.1186/s12885-020-6516-1

**Published:** 2020-01-15

**Authors:** Claire M. Connell, Rebecca Brais, Hayley Whitaker, Sara Upponi, Ian Beh, Jane Risdall, Pippa Corrie, Tobias Janowitz, Duncan I. Jodrell

**Affiliations:** 1 Addenbrooke’s Hospital, Cambridge University Hospitals NHS Foundation Trust, Cambridge, CB2 0QQ UK; 2 Department of Oncology, CRUK Cambridge Institute, Li Ka Shing Centre, University of Cambridge, Cambridge, CB2 0RE UK; 3 Research Department for Tissue & Energy, Division of Surgery & Interventional Science, University College London, Charles Bell House, 43-45 Foley Street, London, W1W 7TS UK

**Keywords:** Cytidine deaminase, Drug metabolism, Gemcitabine, Pancreatic adenosquamous carcinoma

## Abstract

**Background:**

Pancreatic adenosquamous carcinoma has a poor prognosis, with limited prospective trial data to guide optimal treatment. The potential impact of drug metabolism on the treatment response of patients with pancreatic adenosquamous carcinoma is largely unknown.

**Case presentation:**

We describe the case of a 51 year old woman with pancreatic adenosquamous carcinoma who, following surgical resection, experienced early disease relapse during adjuvant gemcitabine therapy. Paradoxically, this was followed by an exceptional response to capecitabine therapy lasting 34.6 months. Strong expression of cytidine deaminase was detected within the tumour.

**Conclusions:**

This case study demonstrates that early relapse during adjuvant chemotherapy for pancreatic adenosquamous carcinoma may be compatible with a subsequent exceptional response to second line chemotherapy, an important observation given the poor overall prognosis of patients with adenosquamous carcinoma. Cytidine deaminase is predicted to inactivate gemcitabine and, conversely, catalyze capecitabine activation. We discuss strong intra-tumoural expression of cytidine deaminase as a potential mechanism to explain this patient’s disparate responses to gemcitabine and capecitabine therapy, and highlight the benefit that may be gained from considering similar determinants of response to chemotherapy in clinical practice.

## Background

Pancreatic adenosquamous carcinoma is estimated to account for fewer than 10% of all pancreatic exocrine malignancies (reviewed in [1]). It is associated with a poor prognosis, with a median survival of 4 months (95% CI, 3–6 months) or 12 months (95% CI, 8–52 months) post-resection in cases of early stage disease [2]. The use of adjuvant chemotherapy is supported by retrospective analyses [2–4], commonly with single agent gemcitabine or 5-fluorouracil according to the evidence base for the treatment of the broader group of pancreatic exocrine malignancies [5, 6] . The optimal management of progressive disease during adjuvant chemotherapy in patients with adenosquamous carcinoma is unknown. We describe the case of a patient with adenosquamous carcinoma experiencing disease progression during adjuvant gemcitabine therapy.

## Case presentation

**Fig. 1:**
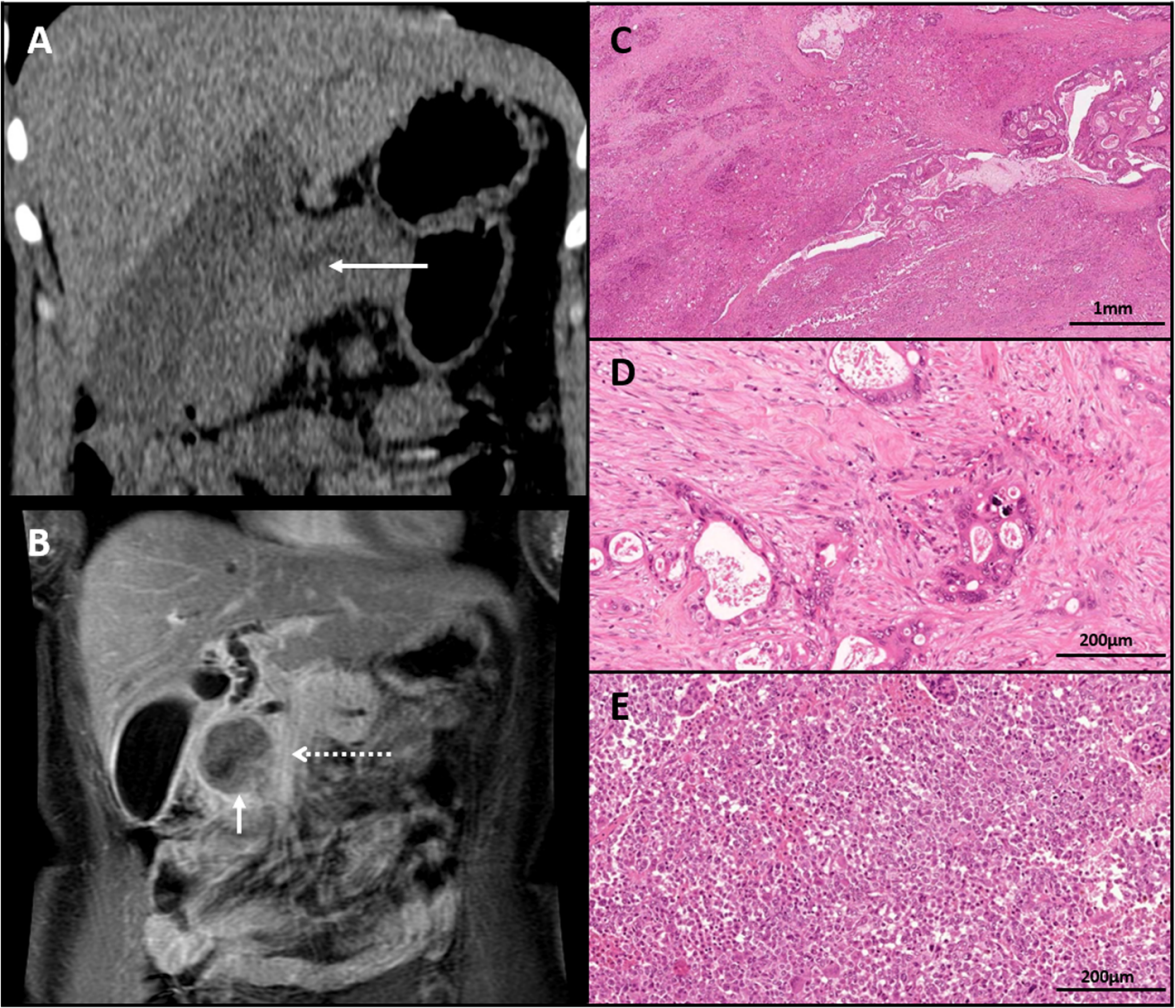
Head of pancreas carcinoma with adenosquamous and undifferentiated components; (**a**) unenhanced CT demonstrating a mass at the head of the pancreas with marked pancreatic duct dilatation (solid arrow); (**b**) Coronal MRI (with gadolinium contrast) demonstrating head of pancreas mass (solid arrow) abutting the adjacent SMV (dashed arrow); (**c**) hematoxylin and eosin stain of head of pancreas tumour from Whipple’s resection demonstrating a biphasic carcinoma with adenosquamous (magnified in (**d**)) and undifferentiated (magnified in (**e**)) components

A previously well 51 year old white Caucasian woman presented with sudden onset right upper quadrant abdominal pain. She took no regular medication and had no medical, surgical or family history of note. Unenhanced computerised tomography (CT) imaging, used due to a pre-existing iodine allergy, identified a 4 cm mass in the head of the pancreas associated with marked pancreatic and mild bile duct dilatation (Fig. 1a). Magnetic resonance (MR) imaging of the liver with gadolinium contrast demonstrated a 4.4 × 3.9 cm mass arising from the inferior head and uncinate process of the pancreas, abutting the superior mesenteric vein (SMV) but with no evidence of liver, peritoneal or mesenteric involvement (Fig. 1b). Positron emission tomography-CT identified metabolically active disease limited to the head of the pancreas. Endoscopic ultrasound guided fine needle aspiration demonstrated poorly differentiated malignancy, with evidence of epithelial and neuroendocrine differentiation with weak chromogranin and focal cytokeratin staining.

At this point the Eastern Cooperative Oncology Group Performance Status (ECOG PS) of the patient was 0. A pancreaticoduodenectomy with SMV resection was performed. Histology confirmed a biphasic carcinoma with adenosquamous and undifferentiated components (Fig. 1c-e); the undifferentiated component was positive for CD56 but negative for other neuroendocrine markers (synpatophysin and chromogranin) and exhibited focal pancytokeratin staining. There was evidence of perineural and lymphovascular space invasion, and 3 out of 25 lymph nodes contained metastatic adenosquamous carcinoma. Resection margins were clear of disease. Staging was confirmed as pT3 pN1 M0 R0 (American Joint Committee on Cancer Stage IIB).

**Fig. 2:**
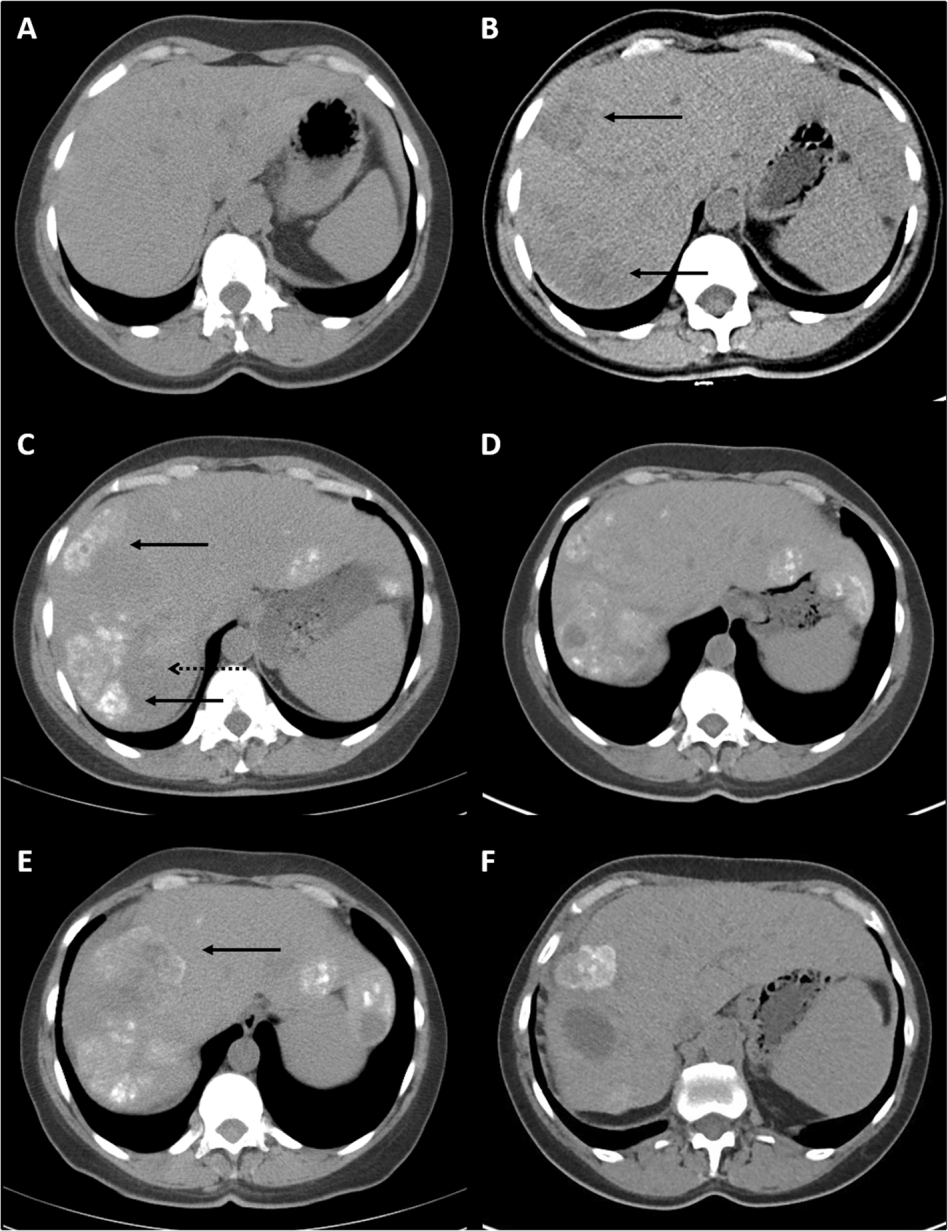
Radiological progression of liver metastases, by unenhanced CT. **a** baseline liver free of metastatic disease; **b** new liver lesions in segments 7 and 8 (solid arrows) 1.8 months into gemcitabine therapy; **c** high attenuation calcification of pre-existing liver lesions (solid arrows) and further low attenuation cystic components (dashed arrow) 13.8 months into capecitabine therapy; **d** stable liver disease 32.3 months into capecitabine therapy; **e** increase in size of segment 8 liver lesion (solid arrow) 34.6 months into capecitabine therapy; **f** stable liver disease 13.3 months into modified FOLFIRINOX therapy

Post-operatively, adjuvant gemcitabine (1000 mg m^− 2^ on days 1, 8 and 15 of a 28-day cycle) chemotherapy was administered as part of the European Study Group for Pancreatic Cancer (ESPAC) 4 Trial (UKCRN ID 4307; ISRCTN96397434). However, after the second cycle of gemcitabine, the patient reported further right upper quadrant pain. ECOG PS dropped to 1 and unenhanced CT confirmed multiple hypodense liver lesions of new onset in keeping with liver metastases (Fig. 2a liver at point of diagnosis; Fig. 2b new liver metastases). Gemcitabine was stopped and palliative capecitabine started (1250 mg m^− 2^ with an 18% dose reduction at patient request to reduce tablet burden). Persistent anaemia, thrombocytopenia and coagulopathy were managed with frequent transfusions of packed red blood cells (PRBC) and oral tranexamic acid.

After 19 weeks of capecitabine therapy ECOG PS returned to 0, and the patient returned to work full time. Haematological indices improved. No further PRBC transfusions were required after 24 weeks and tranexamic acid was stopped. Palmar-plantar erythrodysesthesia, 14.6 months into capecitabine therapy, prompted a switch to a 2 week on, 2 week off treatment schedule which was well tolerated. Restaging CTs after 13.8 and 32.3 months of capecitabine therapy confirmed stable, measurable disease, but with marked calcification of liver metastases, indicative of disease response (Fig. 2c, d). After 34.6 months (41 cycles) of capecitabine monotherapy, the patient reported recurrent right upper quadrant pain. A CT confirmed progressive disease (Fig. 2e) with an increased soft tissue component surrounding the pre-existing calcification within segment 8, in keeping with progressive disease.

Capecitabine was discontinued and FOLFIRINOX chemotherapy (5-FU 400 mg m^− 2^ bolus followed by 2400 mg m^− 2^ infusion over 46 h; irinotecan 180 mg m^− 2^; oxaliplatin 85 mg m^− 2^) started, with granulocyte-colony stimulating factor (GCSF) support. Due to peripheral neurotoxicity, the oxaliplatin dose was reduced by 20% for cycle 7 and discontinued from cycle 8. The patient completed 12 cycles of the reduced intensity chemotherapy. An end of treatment CT demonstrated stable partially calcified liver metastases, and this was followed by a treatment break. Surveillance CT 13.3 months after initiating FOLFIRINOX and 4 years 5.3 months after initial presentation, confirmed stable disease (Fig. 1f).

**Fig. 3:**
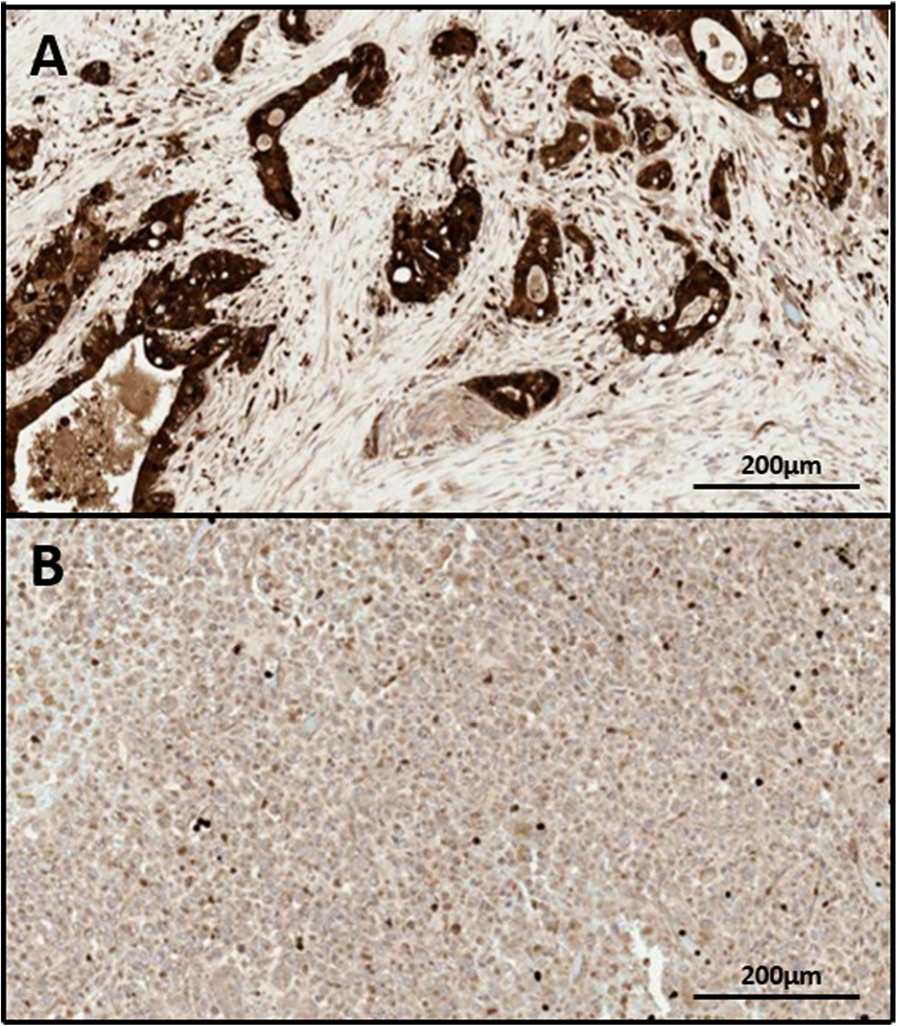
CDA protein expression within adenosquamous and undifferentiated tumour components. Formalin-fixed paraffin-embedded sections of adenosquamous (**a**) and undifferentiated (**b**) tumour components were stained with a polyclonal rabbit anti-CDA antibody (Epitomics) on a Leica BOND-MAX Autostainer (Leica Biosystems, UK), with heat-induced epitope retrieval with pH 6.0 citrate-based solution (ER1, Leica Biosystems, UK)

To better understand the treatment responses observed and to explore future treatment options, tumor targeted DNA sequencing, tumour CDA expression, and whole blood CDA activity were analyzed. Genetic analysis identified a G12D mutation in *KRAS*, a truncating mutation in *TP53*, but no *BRCA1*/ *BRCA2* mutations or currently putative “druggable” mutations [7]. Strong expression of CDA was detected within the glandular and, to a lesser extent, the undifferentiated components of the tumor by immunohistochemistry (Fig. 3a, b). Whole blood CDA activity via spectrophotometry was found to be 6.72 UA/mg protein, below the median CDA activity level observed in this patient population ([8] and unpublished data).

## Discussion and conclusions

The poor prognosis of patients with pancreatic adenosquamous carcinoma may impact on the decision to consider second line chemotherapy. In the case described, despite early relapse during adjuvant gemcitabine therapy, the patient proceeded to have a 34.6 month remission on second line capecitabine. This is an exceptional response. To our knowledge, this is the first documented case of a patient with adenosquamous cancer having an exceptional response to second line treatment, in the context of early disease relapse.

CDA mediates the inactivation of gemcitabine [9] and, conversely, the activation of capecitabine [10]. Relative to the normal pancreas, it has been reported that CDA is overexpressed in pancreatic cancer [11]. Both circulating CDA activity [12, 13], and intra-tumoural expression of CDA [8, 14, 15], varies between patients and may impact on the response of patients to gemcitabine and / or capecitabine therapy. Whereas low circulating CDA activity has been associated with a better response to gemcitabine treatment in patients with pancreatic cancer in two studies [12, 13], this was not the case in a subsequent multicentre prospective trial with 120 patients treated with gemcitabine [16]. For intra-tumoural CDA expression, preclinical data have identified strong expression to be associated with reduced response to gemcitabine [14] and increased response to capecitabine [15]. Clinical data investigating the impact of intra-tumoural CDA activity are lacking, although data from a recent study of 105 patients with pancreatic cancer do suggest that intra-tumoural CDA expression may be predictive of response to sequential, but not concomitant, therapy with nab-paclitaxel and gemcitabine in the first line setting [8]. Germline CDA single nucleotide polymorphisms have also been associated with CDA activity and / or toxicity or survival with gemcitabine based therapy [17–19]. However, CDA single nucleotide polymorphisms are detected in only a subset of patients with reduced CDA activity, supportive of other regulatory mechanisms determining CDA expression and activity (reviewed in [20]). In this case report, we identify strong intra-tumoural expression of CDA as a potential contributor to the patient’s disparate responses to these two chemotherapeutic agents. Importantly, whole blood CDA activity was not elevated, indicating that increased CDA activity was not systemic, and capecitabine was well tolerated. Similar consideration, and reporting, of how intra-tumoural drug metabolism may impact on responses to chemotherapy will facilitate our understanding of these responses and our treatment decisions in the future.

The choice of adjuvant chemotherapy for pancreatic adenosquamous cancer has, to date, been largely guided by trials conducted almost exclusively in patients with pancreatic adenocarcinoma [21–24]. Data from the ESPAC-4 trial suggest that combination therapy with gemcitabine and capecitabine may offer further survival benefit over single agent [23]. Conversely, retrospective analyses restricted to pancreatic adenosquamous cancer have suggested that this subgroup may be most responsive to combination therapy with platinum agents [3, 4]. Whilst evidence supporting the optimal adjuvant regimen for pancreatic adenosquamous tumors remains limited by the relatively low incidence of the disease, this case report demonstrates that early relapse on one regimen may be compatible with a subsequent exceptional response to second line chemotherapy, particularly when a biochemical rationale exists for the failure of initial chemotherapy.

## Data Availability

The data generated and / or analyzed during this study are included in this published article.

## Abbreviations

CDA: Cytidine deaminase
CT: Computerised tomography
ECOG PS: Eastern Cooperative Oncology Group Performance Status
FOLFIRINOX: Fluorouracil, irinotecan, oxaliplatin
GCSF: Granulocyte-colony stimulating factor
MR: Magnetic resonance
PRBC: Packed red blood cells
SMV: Superior mesenteric vein

## References

[1] Borazanci E, Millis S, Korn R, Han H, Whatcott C, Gatalica Z (2015). Adenosquamous carcinoma of the pancreas: molecular characterization of 23 patients along with a literature review. World J Gastrointest Oncol.

[2] Katz MHG, Taylor TH, Al-Refaie WB, Hanna MH, Imagawa DK, Anton-Culver H (2011). Adenosquamous versus adenocarcinoma of the pancreas: a population-based outcomes analysis. J Gastrointest Surg.

[3] Wild AT, Dholakia AS, Fan KY, Kumar R, Moningi S, Rosati LM (2015). Efficacy of platinum chemotherapy agents in the adjuvant setting for adenosquamous carcinoma of the pancreas. J Gastrointest Oncol.

[4] De Souza AL, Saif MW (2014). Platinum-based therapy in adenosquamous pancreatic cancer: experience at two institutions. JOP..

[5] Khorana AA, Mangu PB, Berlin J, Engebretson A, Hong TS, Maitra A (2016). Potentially curable pancreatic Cancer: American Society of Clinical Oncology clinical practice guideline. J Clin Oncol.

[6] Ducreux M, Cuhna AS, Caramella C, Hollebecque A, Burtin P, Goéré D (2015). Cancer of the pancreas: ESMO Clinical Practice Guidelines for diagnosis, treatment and follow-up. Ann Oncol Off J Eur Soc Med Oncol.

[7] Waddell N, Pajic M, Patch AM, Chang DK, Kassahn KS, Bailey P (2015). Whole genomes redefine the mutational landscape of pancreatic cancer. Nature..

[8] Corrie P, Qian W, Gopinathan A, Williams M, Brais R, Valle JW (2017). Strong tumour cytidine deaminase (CDA) staining predicts for improved survival associated with sequential nab-Paclitaxel (nabP) and gemcitabine (GEM) chemotherapy as first line treatment of patients (pts) with metastatic pancreatic adenocarcinoma (mPDAC). Ann Oncol.

[9] Abbruzzese JL, Grunewald R, Weeks EA, Gravel D, Adams T, Nowak B (1991). A phase I clinical, plasma, and cellular pharmacology study of gemcitabine. J Clin Oncol.

[10] Miwa M, Ura M, Nishida M, Sawada N, Ishikawa T, Mori K (1998). Design of a novel oral fluoropyrimidine carbamate, capecitabine, which generates 5-fluorouracil selectively in tumours by enzymes concentrated in human liver and cancer tissue. Eur J Cancer.

[11] Zauri M, Berridge G, Thézénas ML, Pugh KM, Goldin R, Kessler BM (2015). CDA directs metabolism of epigenetic nucleosides revealing a therapeutic window in cancer. Nature..

[12] Serdjebi C, Seitz J-F, Ciccolini J, Duluc M, Norguet E, Fina F (2013). Rapid deaminator status is associated with poor clinical outcome in pancreatic cancer patients treated with a gemcitabine-based regimen. Pharmacogenomics..

[13] Bengala C, Guarneri V, Giovannetti E, Lencioni M, Fontana E, Mey V (2005). Prolonged fixed dose rate infusion of gemcitabine with autologous haemopoietic support in advanced pancreatic adenocarcinoma. Br J Cancer.

[14] Frese KK, Neesse A, Cook N, Bapiro TE, Lolkema MP, Jodrell DI (2012). Nab-paclitaxel potentiates gemcitabine activity by reducing cytidine deaminase levels in a mouse model of pancreatic cancer. Cancer Discov.

[15] Morita T, Matsuzaki A, Kurokawa S, Tokue A (2003). Forced expression of cytidine deaminase confers sensitivity to capecitabine. Oncology..

[16] Serdjebi C, Gagnière J, Desramé J, Fein F, Guimbaud R, François E (2015). FFCD-1004 clinical trial: Impact of cytidine deaminase activity on clinical outcome in gemcitabine-monotherapy treated patients. PLoS One.

[17] Sugiyama E, Kaniwa N, Kim SR, Kikura-Hanajiri R, Hasegawa R, Maekawa K (2007). Pharmacokinetics of gemcitabine in Japanese cancer patients: the impact of a cytidine deaminase polymorphism. J Clin Oncol.

[18] Ueno H, Kaniwa N, Okusaka T, Ikeda M, Morizane C, Kondo S (2009). Homozygous CDA*3 is a major cause of life-threatening toxicities in gemcitabine-treated Japanese cancer patients. Br J Cancer.

[19] Zeng H, Yu H, Lu L, Jain D, Kidd MS, Saif MW (2011). Genetic effects and modifiers of radiotherapy and chemotherapy on survival in pancreatic cancer. Pancreas..

[20] Mercier C, Evrard A, Ciccolini J (2007). Genotype-based methods for anticipating gemcitabine-related severe toxicities may lead to false-negative results. J Clin Oncol.

[21] Neoptolemos JP, Dunn JA, Stocken DD, Almond J, Link K, Beger H (2001). Adjuvant chemoradiotherapy and chemotherapy in resectable pancreatic cancer: a randomised controlled trial. Lancet..

[22] Neoptolemos JP, Stocken DD, Bassi C, Ghaneh P, Cunningham D, Goldstein D (2010). Adjuvant chemotherapy with fluorouracil plus folinic acid vs gemcitabine following pancreatic cancer resection: a randomized controlled trial. JAMA - J Am Med Assoc.

[23] Neoptolemos JP, Palmer DH, Ghaneh P, Psarelli EE, Valle JW, Halloran CM (2017). Comparison of adjuvant gemcitabine and capecitabine with gemcitabine monotherapy in patients with resected pancreatic cancer (ESPAC-4): a multicentre, open-label, randomised, phase 3 trial. Lancet..

[24] Oettle H, Post S, Neuhaus P, Gellert K, Langrehr J, Ridwelski K (2007). Adjuvant chemotherapy with gemcitabine vs observation in patients undergoing curative-intent resection of pancreatic cancer: a randomized controlled trial. JAMA.

